# Prognostic value of monitoring tumour markers CA 15-3 and CEA during fulvestrant treatment

**DOI:** 10.1186/1471-2407-6-81

**Published:** 2006-03-26

**Authors:** Rupert Bartsch, Catharina Wenzel, Ursula Pluschnig, Dagmar Hussian, Ursula Sevelda, Gabriela Altorjai, Gottfried J Locker, Robert Mader, Christoph C Zielinski, Guenther G Steger

**Affiliations:** 1 Department of Internal Medicine I, Division of Oncology, Medical University of Vienna, Vienna, Austria

## Abstract

**Background:**

At many centres tumour markers are used to detect disease recurrence and to monitor response to therapy in patients with advanced disease, although the real value of serial observation of marker levels remains disputed. In this study, we evaluated the prognostic value of tumour markers for predicting response (partial response [PR], stable disease [SD] ≥ 6 months), *de novo *disease progression (PD) and secondary PD in patients receiving fulvestrant ('Faslodex') 250 mg/month for the treatment of metastatic breast cancer (MBC).

**Methods:**

Changes in cancer antigen 15–3 (CA 15-3) and carcinoembryonic antigen (CEA) were prospectively monitored (monthly) and were also evaluated for the 3 months preceding secondary PD. Data from 67 patients with previously treated MBC participating in a Compassionate Use Programme were analysed.

**Results:**

In patients with a PR (*n *= 7 [10.4%]), a non-significant increase in CA 15-3 occurred during the first 6 months of treatment; CEA was significantly reduced (*P *= 0.0165). In patients with SD ≥ 6 months (*n *= 28 [41.8%]), both CA 15-3 (*P *< 0.0001) and CEA (*P *= 0.0399) levels increased significantly after 6 months treatment. In those experiencing *de novo *PD (*n *= 32 [47.8%]), CA 15-3 increased significantly (*P *< 0.0001) after 4 months; CEA also increased significantly (*P *= 0.0002) during the same time period. Both CA 15-3 (*P *< 0.0001) and CEA (*P *< 0.0001) increased significantly in the 3 months preceding secondary PD.

**Conclusion:**

CA 15-3 increases in patients progressing on fulvestrant but may also increase in those experiencing clinical benefit; this should not be taken as a sign of PD without verification. Overall, both CA 15-3 and CEA appear to be poor prognostic markers for determining progression in patients receiving fulvestrant.

## Background

Tumour markers are often used to detect disease recurrence after primary treatment for cancer [1,2] and to monitor response to therapy in patients with advanced disease [3]. In breast cancer, the most widely adopted combination of tumour markers are carcinoembryonic antigen (CEA) and mucin (MUC1), commonly detected as cancer antigen (CA) 15-3 [4]. Of these two markers, CA 15-3 is generally regarded as the most specific and sensitive. From as early as 1978 investigators were examining the prognostic value of CEA in patients receiving endocrine therapy for the treatment of metastatic breast cancer (MBC) [5], but the value of tumour markers in monitoring response to fulvestrant ('Faslodex') treatment has not previously been evaluated.

It is commonly believed that an increase in tumour marker levels may be a sign of *de novo *disease progression (PD), thus signalling that a change in therapy may be necessary [6]. A recent prospective study found that the changes in serum tumour marker levels after the start of therapy correlate significantly with therapy response; and a greater than 20% reduction in marker levels was a favourable predictive factor for time to progression during systemic treatment [7]. However, in 1996 Sonoo suggested that serial levels of tumour markers taken during therapy might not always correlate with therapy response [8]. This group observed an initial increase and subsequent decrease in tumour marker levels in up to one-third of patients experiencing clinical benefit (CB). One reason for this spike phenomenon might be the possible tumour flair associated with the partial agonistic properties of selective estrogen receptor modulators (SERMs), which however was not observed with fulvestrant yet. A possible alternative explanation for this spike is that it results from the destruction of tumour cells on successful treatment, similar to the increase in muscle enzymes observed following myocardial infarction. Another group recently observed that changes in serial tumour marker levels do not allow prediction of bone scan results [9]. Thus the real value of serial observation of marker levels remains somewhat disputed.

Fulvestrant is the first of a new type of oestrogen receptor (ER) antagonist to be licensed for the treatment of advanced breast cancer [10,11]. Fulvestrant binds, blocks and degrades the ER, thus decreasing cellular levels of the ER and minimising the transcription of oestrogen- and progesterone-dependent genes [12,13]. Two large PhaseIII trials have shown that fulvestrant is at least as effective as anastrozole in the treatment of patients with advanced breast cancer progressing on/after antioestrogen therapy [14-17]. It has also been shown to have similar efficacy as tamoxifen in the first-line treatment of patients with ER-positive and/or progesterone receptor (PgR)-positive disease [18]. Here we evaluated the prognostic value of tumour markers at predicting response and secondary progression during fulvestrant therapy.

## Methods

All data were collected at the Department of Internal Medicine I, Division of Oncology at the Medical University of Vienna, Vienna, Austria. The study was performed in accordance with the ethical regulations of the Medical University of Vienna.

Criteria for inclusion were as follows: Presence of at least one measurable lesion, Karnofsky performance score ≥ 70%, life expectancy of >3 months, adequate organ function as defined by WBC count ≥ 3500/μl, platelet count ≥ 100 000/μl, hematocrit ≥ 30% and serum bilirubin and creatinine ≤ 1.25 × upper limit of the institution's normal range, and informed consent. We included patients with marker levels below the cut-offs at the initiation of treatment or dropping below the cut-offs during the course of fulvestrant treatment. However, patients were excluded from this study if they never experienced an elevation of marker levels above cut-offs on treatment.

Fulvestrant was administered monthly as a single 250 mg intramuscular injection [19] as part of a compassionate use programme (supported by AstraZeneca) until PD or other event necessitating withdrawal. All patients were postmenopausal women with ER-positive and/or PgR-positive MBC for whom at least one prior endocrine therapy had failed, either as adjuvant treatment or as the treatment of advanced disease. Many had also received chemotherapy in the adjuvant and/or advanced disease settings. Table 1 lists the characteristics of all patients included.

### Assessments

Tumour response was assessed every 3 months using UICC criteria. Partial response (PR) was defined as a ≥ 25% reduction in the sum of products of the diameters of all measurable lesions, no increase in lesion size and no new lesions. Stable disease (SD) was defined as a <25% decrease or < 25% increase in the sum of products of the diameters of all measurable lesions without the appearance of new lesions (maintained for a minimum of 6 months). PD was defined as a >25% increase in lesion size or the appearance of new lesions. Tumour response was assessed via computed tomography (CT) scans of the thorax and the abdomen and, where appropriate, also using magnetic resonance imaging or bone scans. An individual patient's response was listed as the best overall response during the course of treatment.

Levels of the tumour markers CA 15-3 and CEA were prospectively monitored on a monthly basis during fulvestrant treatment (at the time of each injection). Patients experiencing SD with fulvestrant were retrospectively split into two groups for the purpose of the analysis of CA 15-3 levels: those experiencing SD ≥ 6 but < 9 months and those experiencing SD ≥ 9 months.

We also prospectively investigated whether a rise in CA 15-3 or CEA levels during the course of therapy could predict secondary progression in patients who had previously experienced CB (PR or SD ≥ 6 months) with fulvestrant. For this, tumour marker levels were analysed over the last 3 months of treatment prior to the final response assessment at which the patient was deemed to be progressing.

CEA and CA 15-3 levels were assessed using the specific CEA and CA 15-3 Elecsys^® ^2010 reagents (Roche Diagnostics AG, Basel, Switzerland) for Elecsys^® ^2010 Disc System (Roche Diagnostics, Basel, Switzerland). Cut-off values of the assay methods were 3.4 μg/l in CEA and 30 kU/l in CA 15-3.

### Statistical analysis

Tumour marker levels (CEA and CA 15-3) were analysed during the first 6 months of therapy with fulvestrant in all patients, and 9 months in patients with CB ≥ 9 months. They were also analysed during the last 3 months of treatment in patients experiencing secondary progression following CB. The Friedman test and GraphPad PRISM Version 4.00 were used to analyse any observed changes and to assess their statistical significance.

As no survival analysis was performed, CB was used as surrogate parameter for the evaluation of the prognostic implication of marker levels.

## Results

### Patients and response

Tumour marker levels and response were analysed in 67 patients receiving fulvestrant treatment between 2002 and 2004. The median age of the patients was 63 years (range 31–83 years). Seven patients (10.5%) experienced a PR with fulvestrant treatment, 28 patients (41.8%) experienced SD ≥ 6 months, while 32 patients (47.8%) had *de novo *PD.

### Tumour marker levels in patients experiencing a PR

In the seven patients experiencing a PR an increase in CA 15-3 levels was observed over the first 4 months of treatment (Table 2); this did not reach statistical significance. Similar observations were noted after 6 months of treatment. CEA levels showed a non-significant decrease after 3 months of therapy (*P *= 0.2103), which reached significance after 6 months (*P *= 0.0165).

### Tumour marker levels in patients experiencing SD ≥ 6 months

**Figure 1:**
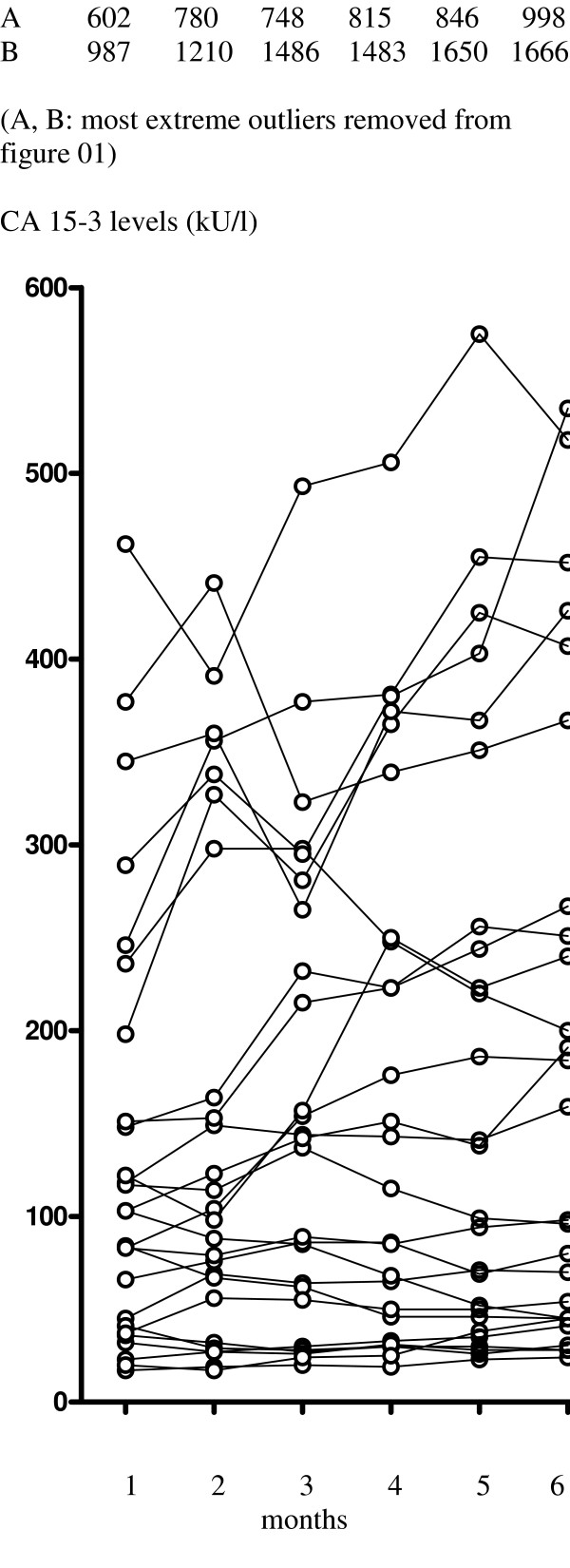
Observed changes in CA 15-3 levels in patients with stable disease ≥ 6 months from baseline to 6th treatment (*n *= 28).

In the 28 patients with SD ≥ 6 months CA 15–3 serial levels increased significantly between baseline and 4 months (*P *= 0.0023) (Table 2; Fig. 1) and were also significantly increased at 6 months (*P *< 0.0001). Further assessments carried out because of this unexpected increase in CA 15-3 levels were retrospectively evaluated in two separate subgroups (SD ≥ 6 but < 9 months [*n *= 14] and SD ≥ 9 months [*n *= 14]). Between baseline and 4 months a significant increase in CA 15-3 levels was observed both in the SD ≥ 6 but < 9 months (*P *= 0.0017) and the SD ≥ 9 months (*P *= 0.0405) subgroups, both of which were maintained at 6 months. In the group of patients experiencing SD ≥ 6 months overall, CEA levels also increased significantly between baseline and 6 months (*P *= 0.0399).

### Tumour marker levels in patients experiencing PD

**Figure 2:**
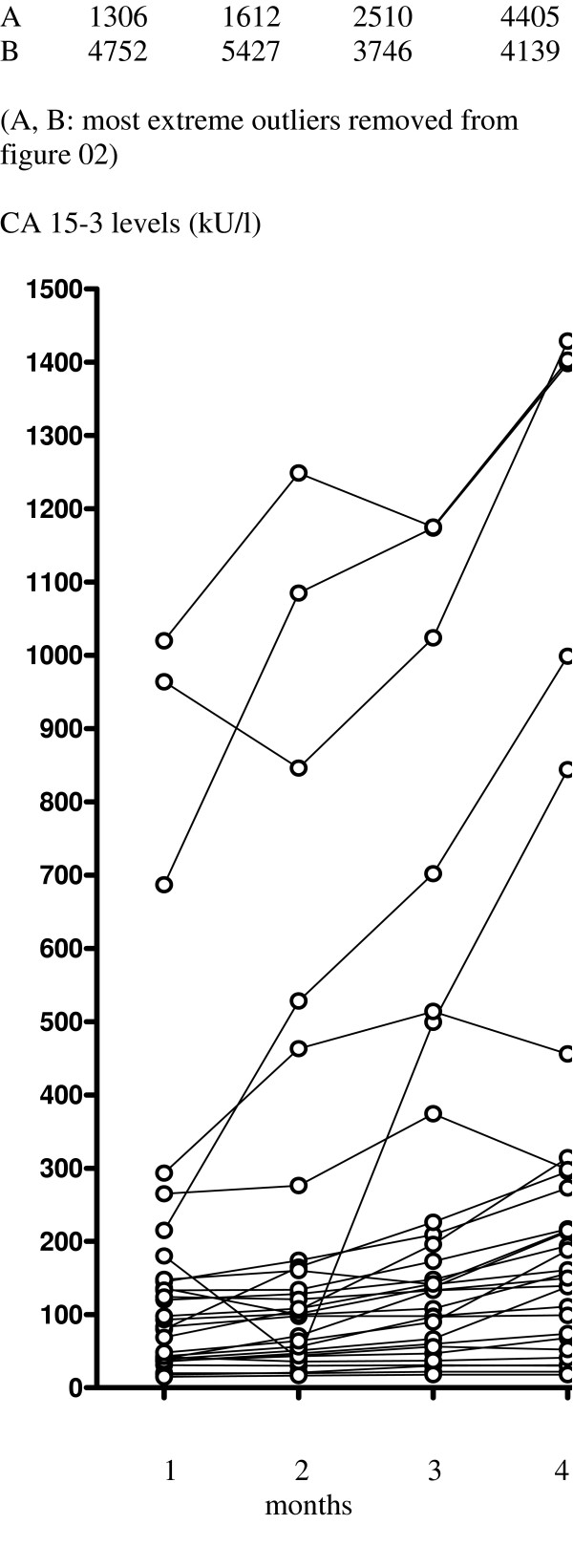
Observed changes in CA 15-3 levels in patients with *de novo *disease progression from baseline to 4th treatment (*n *= 32).

In the 32 patients experiencing *de novo *PD a significant increase (*P *< 0.0001) in CA 15-3 levels was noted between baseline and 4 months (Table 2; Fig. 2). CEA levels also showed a significant increase (*P *= 0.0002) over the same period.

### Tumour marker levels in those experiencing secondary progression

**Figure 3:**
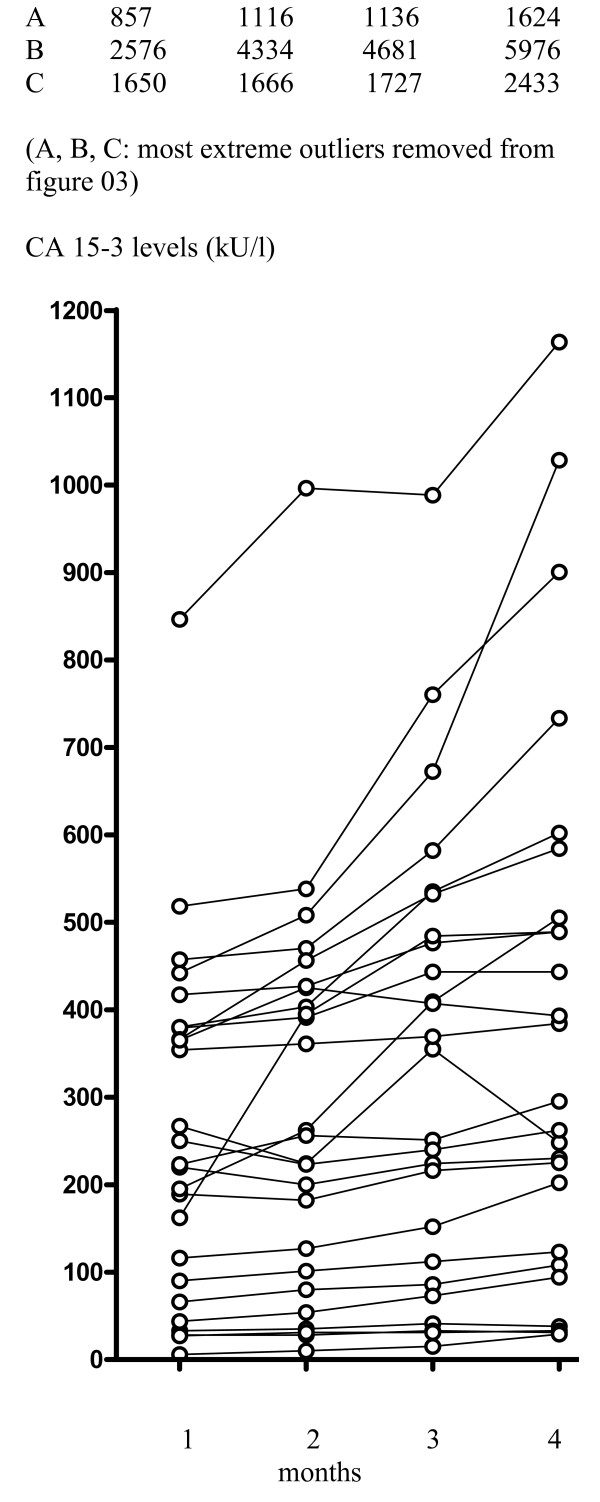
Observed changes in CA 15-3 levels in patients 3 months prior to secondary progression after experiencing clinical benefit with fulvestrant treatment (*n *= 28).

In patients experiencing CB followed by secondary progression (*n *= 28), a significant increase in CA 15-3 levels was observed during the last 3 months of treatment (*P *< 0.0001) (Fig. 3). CEA levels also increased significantly (*P *< 0.0001) during this time.

## Discussion

Our group was amongst the first in continental Europe to treat patients with fulvestrant as part of a compassionate use programme supported by AstraZeneca. In our institution it is common practice to evaluate tumour marker levels following each month of palliative therapy. During informal analysis of such data in patients receiving fulvestrant, potential differences in tumour marker kinetics were noted within the subgroup of patients responding to therapy and thus a detailed investigation was carried out.

In this study a trend towards increased CA 15-3 levels was noted during the first 3 months in patients responding to fulvestrant treatment. This trend became stronger over time, but still failed to reach statistical significance after 6 months of treatment. This result was unexpected since these patients were responding to fulvestrant therapy. The only occasion in which CEA appeared to have a higher predictive value than CA 15-3 was in the subgroup of patients experiencing a PR, where the observed decrease in CEA levels attained statistical significance after 6 months.

In the group of patients experiencing SD we found a significant increase in CA 15-3 levels at both 3 and 6 months. This was observed both in patients with SD ≥ 6 months but < 9 months and in those experiencing SD ≥ 9 months, thus this observation is unlikely to result from a bias caused by a lead-time effect to actual PD. There are several possible explanations for this early increase in tumour marker levels. First, it may be as a result of the delay in reaching steady-state levels with fulvestrant treatment [20], although this appears unlikely as these patients were already experiencing CB with fulvestrant. Nonetheless it seems reasonable to ask whether a loading-dose regimen of fulvestrant may be appropriate and this type of dosing is under investigation in ongoing clinical trials [21]. However, as initial increases in CEA and CA 15-3 have also been observed in patients experiencing CB with other breast cancer therapies [8,22-25], the dose of fulvestrant seems unlikely to be the cause. One explanation is that the increase in tumour markers may result from increased tumour degradation in response to fulvestrant treatment. These data may suggest that, in the absence of radiological detection of PD, fulvestrant treatment should be continued beyond 3 months before response to therapy is assessed. Marker levels in patients with *de novo *PD increased steadily during fulvestrant treatment as may be expected in the presence of non-responsive disease. As tumour marker levels may rise in both responding and non-responding patients during the first few months of fulvestrant treatment, it may be that an increase in markers following a period of stabilisation may be more predictive of disease progression.

The data presented here suggest that if increased CA 15-3 levels are observed after the first 3 months of fulvestrant treatment this should not be taken as a sign of PD without radiological verification. On the contrary, such an increase may also be observed in patients gaining CB from treatment. Consequently, our results demonstrate that it is inappropriate to change therapy purely based on increased tumour marker levels, as some patients may still be benefiting from fulvestrant treatment.

In our study CEA levels were found to decrease significantly after 6 months in patients experiencing a PR and to increase significantly in those with SD. Significant rises in CEA were also observed in patients experiencing *de novo *PD.

The predictive value of tumour markers in signalling secondary progression was also prospectively assessed. We found that both CA 15-3 and CEA levels increased significantly during the last 3 months of treatment and so these markers may be valuable in predicting secondary progression. However, both the CEA and CA 15-3 data need to be treated with some caution because of the small number of patients in this study.

## Conclusion

In conclusion, our results suggest that increased CA 15-3 levels may be observed in patients experiencing a PR with fulvestrant, however, increased CA 15-3 levels are also possible in patients experiencing either SD or PD. Thus it is important that such an increase is not taken as a definite sign of *de novo *progression without radiological verification. An increase in CA 15-3 or CEA levels may also precede secondary progression in patients who had previously gained CB from fulvestrant treatment. Overall, both CA 15-3 and CEA appear to be poor prognostic markers for determining progression in patients receiving fulvestrant.

## Abbreviations

CEA carcinoembryonic antigen

CA 15-3 cancer antigen 15-3

MBC metastatic breast cancer

PR partial remission

SD stable disease

PD progressive disease

CB clinical benefit (PR or SD ≥ 6 months)

## Competing interests

The author(s) declare that they have no competing interests.

## Authors' contributions

RB contributed the idea for this trial, participated in the design and drafted the manuscript, CW participated in the statistical analysis and helped drafting the manuscript, UP participated in the design of the study, DH and US both participated in the collection of patient data, GA helped in the design and coordination of the study, GL revised the manuscript critically, RM assisted in the statistical analysis, CZ and GS were important in the design of the study, the coordination and helped to draft the manuscript.

All authors have read and approved the final manuscript.

## Pre-publication history

The pre-publication history for this paper can be accessed here:



## Figures and Tables

**Table 1:** Patient characteristics

Characteristics	Patients
Entered	67
Age (years)	
Median (range)	62 years (range 39 – 83 y)
Estrogens receptor positive	62 (92.5%)
Progesterone receptor positive	42 (62.7%)
Her2 positive	10 (14.9%)
Adjuvant chemotherapy	27 (40.3%)
Adjuvant endocrine therapy	36 (53.7%)
Palliative chemotherapy	44 (65.7%)
Fulvestrant treatment	
1^st ^line	3 (4.5%)
2^nd ^line	27 (40.3%)
3^rd ^line	28 (41.8%)
4^th ^line	9 (13.4%)
Prior endocrine treatment	
Tamoxifen	17 (25.4%)
Anastrozole/Letrozole	59 (88.1%)
Exemestane	32 (47.8%)
MPA	1 (1.5%)
Goserelin	1 (1.5%)
Metastatic sites	
Lung	19
Liver	20
Bones	44
Lymph nodes	15
Soft tissue	19
Skin	10
Other	1
More than one metastatic site	40 (59.7%)
Number of met. Sites	
Median (range)	2 (range 1 – 4)

**Table 2:** Mean tumour marker levels during fulvestrant treatment

Patient response	mean marker levels (+/- SD) (month)								
	1	2	3	4	5	6	7	8	9
				*P*-values (1→4 months)		*P*-values (1→6 months)			
PR (*n *= 7)									
CA 15-3	144.00	128.54	156.87	178.16	170.19	197.01			
(kU/l)	+/-171.75	+/-164.90	+/-194.54	+/-227.42	+/-256.43	+/-270.54			
				N.S.		N.S.			
CEA	5.31	4.17	3.87	3.81	3.46	4.06			
(μg/l)	+/-3.96	+/-2.82	+/-2.61	+/-2.54	+/-2.78	+/-2.95			
				N.S.		0.0165			
SD ≥ 6 months (*n *= 28)									
CA 15-3	184.68	214.00	226.54	240.64	269.05	268.61			
(kU/l)	/-214.08	+/-262.54	+/-297.58	+/-305.39	+/-352.19	+/-338.27			
				0.0023		< 0.0001			
CEA	52.59	43.63	43.49	58.01	55.28	72.70			
(μg/l)	+/-201.99	+/-153.74	+/-153.73	+/-224.58	+/-203.94	+/-307.61			
				N.S.		0.0399			
SD ≥ 6 < 9 months (*n *= 14)									
CA 15-3	205.21	232.21	265.50	281.07	302.71	309.04			
(kU/l)	+/-256.19	+/-309.81	+/-378.08	+/-378.57	+/-424.97	+/-429.82			
				0.0017		0.0002			
SD ≥ 9 months (*n *= 14)									
CA 15-3	164.14	195.79	185.57	200.21	214.50	229.07	209.55	258.61	263.29
(kU/l)	+/-169.24	+/-215.55	+/-194.18	+/-216.47	+/-229.92	+/-263.48	+/-226.84	+/-283.62	+/-292.21
				0.0405		0.0146			
*De novo *PD (*n *= 32)									
CA 15-3	259.91	419.41	441.16	580.31					
(kU/l)	+/-419.29	+/-994.42	+/-790.95	+/-1052.69					
				< 0.0001					
CEA	140.61	192.37	199.98	250.09					
(μg/l)	+/-652.01	+/-902.53	+/-907.67	+/-1087.32					
				0.0002					
Secondary PD (*n *= 28)									
CA 15-3	411.20^a^	510.67^b^	572.50^c^	698.72^d^					
(kU/l)	+/-541.50	+/-834.03	+/-891.17	+/-1164.36					
				< 0.0001					
CEA	56.32 ^a^	53.46 ^b^	69.99 ^b^	110.32 ^d^					
(μg/l)	+/-224.80	+/-203.92	+/-269.53	+/-473.03					
				< 0.0001					

PR, partial response; SD, stable disease; PD, disease progression; NS, not statistically significant; ^a^2 months before PD; ^b^1 months before PD; ^c^PD; ^d^1 month after PD
